# First Isolation and Direct Evidence for the Existence of Large Small-Mammal Reservoirs of *Leptospira* sp. in Madagascar

**DOI:** 10.1371/journal.pone.0014111

**Published:** 2010-11-24

**Authors:** Soanandrasana Rahelinirina, Albertine Léon, Rudy A. Harstskeerl, Natacha Sertour, Ahmed Ahmed, Claudine Raharimanana, Elisabeth Ferquel, Martine Garnier, Loïc Chartier, Jean-Marc Duplantier, Lila Rahalison, Muriel Cornet

**Affiliations:** 1 Plague Unit, Pasteur Institute, Antananarivo, Madagascar; 2 Frank Duncombe Laboratory, IFR 146 ICORE, Caen, France; 3 WHO/FAO/OIE and National Leptospirosis Reference Centre, KIT Biomedical Research, Amsterdam, The Netherlands; 4 National Reference Centre for *Borrelia*, Pasteur Institute, Paris, France; 5 Epidemiology Unit, Pasteur Institute, Paris, France; 6 IRD, UMR CBGP (INRA/IRD/Cirad/Montpellier SupAgro), Montferrier-sur-Lez, France; 7 Parasitology-Mycology Laboratory, CHU Grenoble, University Joseph Fourier, Grenoble, France; St. Petersburg Pasteur Institute, Russian Federation

## Abstract

**Background:**

Leptospirosis has long been a major public health concern in the southwestern Indian Ocean. However, in Madagascar, only a few, old studies have provided indirect serological evidence of the disease in humans or animals.

**Methodology/Principal Findings:**

We conducted a large animal study focusing on small-mammal populations. Five field trapping surveys were carried out at five sites, from April 2008 to August 2009. Captures consisted of *Rattus norvegicus* (35.8%), *R. rattus* (35.1%), *Mus musculus* (20.5%) and *Suncus murinus* (8.6%). We used microbiological culture, serodiagnosis tests (MAT) and real-time PCR to assess *Leptospira* infection. *Leptospira* carriage was detected by PCR in 91 (33.9%) of the 268 small mammals, by MAT in 17 of the 151 (11.3%) animals for which serum samples were available and by culture in 9 of the 268 animals (3.3%). Rates of infection based on positive PCR results were significantly higher in Moramanga (54%), Toliara (48%) and Mahajanga (47.4%) than in Antsiranana (8.5%) and Toamasina (14%) (p = 0.001). The prevalence of *Leptospira* carriage was significantly higher in *R. norvegicus* (48.9%), *S. murinus* (43.5%) and *R. rattus* (30.8%) than in *M. musculus* (9.1%) (p<0.001). The MAT detected antibodies against the serogroups Canicola and Icterohaemorrhagiae. Isolates were characterized by serology, *secY* sequence-based phylogeny, partial sequencing of *rrs*, multi-locus VNTR analysis and pulsed field gel electrophoresis. The 10 isolates obtained from nine rats were all identified as species *L. interrogans* serogroup Canicola serovar Kuwait and all had identical partial *rrs* and *secY* sequences.

**Conclusions/Significance:**

We present here the first direct evidence of widespread leptospiral carriage in small mammals in Madagascar. Our results strongly suggest a high level of environmental contamination, consistent with probable transmission of the infection to humans. This first isolation of pathogenic *Leptospira* strains in this country may significantly improve the detection of specific antibodies in human cases.

## Introduction

Leptospirosis is a zoonosis of global importance caused by a spirochete of the genus *Leptospira*. In several mammalian species, chronic infection of the renal tubules leads to the shedding of the bacterium in the urine. Rodents, insectivores, cattle and swine are considered to be the main reservoirs of this spirochete. Rodents, in particular, are very efficient maintenance hosts because they remain healthy during lifelong renal carriage. They therefore play a key role in environmental contamination. Transmission to humans is mostly indirect, through contact with an infected environment [1], [2]. The clinical presentation of leptospirosis varies, with an early stage characterized by a non specific flu-like syndrome, followed by progression to multiple-organ failure and death in 5 to 15% of patients [1]–[4]. Diagnosis is confirmed by isolation of the bacterium from body fluids or detection of its DNA by polymerase chain reaction (PCR) during the acute phase or of specific antibodies during convalescence.

As bacterial survival is favored by tropical conditions, such as warm fresh water, leptospirosis is considered to be highly endemic in tropical and subtropical regions [1]. In the southwestern Indian Ocean and Africa, leptospirosis has long been a major public health concern [5]–[9]. In the islands close to Madagascar, reported incidence rates range from 5/100,000 and 9/100,000 in La Réunion and Mayotte, respectively, to 101/100,000 in the Seychelles [10], [11]. The environmental and socioeconomic conditions of Madagascar, with its tropical climate, rice and sugar cane agriculture, livestock farming, slums and presence of the notorious commensal rodents *Rattus norvegicus* (brown rat) and *R. rattus* (black rat), appear favorable for leptospirosis transmission [12]. However, although the conditions in Madagascar resemble those of the other nearby islands, the disease has rarely been reported in either humans or animals, with diagnosis based solely on indirect evidence obtained through antibody detection. Half a century ago, a case of local human infection was reported in a patient from Antalaha (on the north-east coast of the island) who presented with fever, icterus, hematuria and neurological disorders. Diagnosis was based on the serological microscopic agglutination test (MAT) [13]. Only one other study, conducted in the Toliara district, detected both human and animal leptospirosis. Silverie *et al.* reported that 51% of patients with clinically suspected disease were seropositive for serogroups Tarassovi, Grippotyphosa, Hebdomadis and Australis, and that the seroprevalence of *Leptospira* was 46% in cattle and 8% in swine [14]. All additional efforts to detect the infection failed to confirm these results. In a survey conducted in Antananarivo on 2646 serum samples from subjects with no symptoms suggestive of leptospirosis, agglutinating antibodies against the serogroups Icterohaemorrhagiae, Grippotyphosa and Canicola were found in only five samples [15]. In a subsequent study, 105 occupationally exposed workers were screened serologically; only one had a low antibody titer [16]. Animal surveys have reported an absence of seropositivity in dogs, sheep, donkeys, horses, cattle and swine from other sites [17]. No pathogenic strains were obtained after bacteriological culture of 55 *R. rattus* and 50 *Pteropus rufus* (Madagascar flying fox) kidneys collected at Marovitsika-Anjiro (100 km north of Antananarivo) [15]. More recently, a PCR method was unable to detect kidney carriage in 115 rats, 50 zebu cattle and 13 pigs from various places [16].

Based on these findings, Madagascar seems to be exceptional among the islands of this region in having a low prevalence of leptospirosis. However, the reasons for this apparent lack of infection remain unclear [15], [16]. We wondered whether this lack of infection reflects the actual situation on the island or whether it can be attributed to the fairly limited investigations of small samples from only a few locations. Moreover, poor conditions for the performance of field studies in this country, together with the cumbersome methods used to confirm the diagnosis (bacteriological culture and MAT) may have hampered such studies in the past. We conducted an extensive animal study, focusing on rodent populations, using microbiological culture, MAT serodiagnosis and a sensitive real-time PCR protocol to investigate the possible maintenance hosts of leptospirosis in Madagascar [18]. We report the first isolation of pathogenic *Leptospira* strains from this country, providing evidence that small animals form a major reservoir in both urban and rural settings on the island.

## Results

### Trapping of small mammals

We captured 268 small mammals in the five study districts: 96 (35.8%) were identified as *Rattus norvegicus*, 94 (35.1%) as *R. rattus*, 55 (20.5%) as *Mus musculus* (the domestic mouse), and 23 (8.6%) as *Suncus murinus* (Asian house shrew). The trapped species differed significantly in terms of their urban/rural distributions and their distributions between districts (p<0.001). *R. rattus* accounted for 63.6% of the small mammals caught in rural areas, whereas *R. norvegicus* was the predominant species in urban areas, accounting for 54.7% of the mammals caught. Two distinct patterns of rodent species distribution as a function of district were observed (Table 1). In Mahajanga and Toliara, *R. norvegicus* was the principal species, accounting for 71.2% and 70.0% of the small mammals caught, respectively. These two sites were also remarkable in that *R. rattus* was absent, even in rural areas. By contrast, at the other three sites, Moramanga, Antsiranana and Toamasina, *R. rattus* was the major species, accounting for 68.0%, 55.9% and 54.0% of all captures, respectively. *M. musculus* accounted for a significantly lower proportion of the animals caught at Mahajanga (6.8%) than at the other four sites (mean of 24.4%; p = 0.003), whereas *S. murinus* accounted for a significantly higher proportion of the animals caught in this district (22% vs a mean of 4.8%; P<0.001) (Table 1).

**Table 1:** Number of small mammals by trapping site and species.

District	Site name/GPS reference	Urban/Rural	Small mammals trapped – n (%)				
			*Rattus norvegicus*	*Rattus rattus*	*Mus musculus*	*Suncus murinus*	All
Moramanga	Ambatosoratra17°54′S 48°51′E	Rural	-	1	-	-	1
	Morarano18°36′S 48°16′E	Urban	1	5	-	2	8
	Ambohibao18°75′S 48°26′E	Rural	2	22	10	1	35
	Ambohibary south18°32′S 48°12′E	Rural	-	6	-		6
	All		3 (6)	34 (68)	10 (20)	3 (6)	50 (100)
Antsiranana	Harbor12°26′S 49°28′E	Urban	5	3	4	-	12
	Andasoa12°27′S 49°29′E	Urban	2	10	12	1	25
	Sakaramy12°44′S 49°26′E	Rural	-	20	2	-	22
	All		7 (11.9)	33 (55.9)	18 (30.5)	1 (1.7)	59 (100)
Mahajanga	Mahajanga city15°72′S 46°32′E	Urban	35	-	4	4	43
	Tsararano Ambony15°70′S 46°29′E	Urban	5	-	-	-	5
	Tsimahajao*16°11′S 46°63′E	Rural	2	-	-	9	11
	All		42 (71.2)	-	4 (6.8)	13 (22)	59 (100)
Toamasina	Harbor18°09′S 49°25′E	Urban	5	-	-	-	5
	Ankirihiry18°08′S 49°24′E	Urban	4	1	7	5	17
	Fanandrana18°16′S 49°14′E	Rural	-	26	1	1	28
	All		9 (18)	27 (54)	8 (16)	6 (12)	50 (100)
Toliara	Harbor23°37′S 43°66′E	Urban	11	-	-	-	11
	Ankiembe23°37′S 43°67′E	Urban	7	-	3	-	10
	Sarodrano23°37′S 43°67′E	Urban	7	-	7	-	14
	Ambalaronde23°37′S 43°67′E	Rural	10	-	5	-	15
	All		35 (70)	-	15 (30)	-	50 (100)
**All**			96 (35.8)	94 (35.1)	55 (20.5)	23 (8.6)	268 (100)

### Carriage of *Leptospira* in small mammals

The results obtained for *Leptospira* detection are detailed in Table 2. Pathogenic *Leptospira* carriage was detected by molecular, serological or culture methods in 33.9% of the 268 small mammals sampled. Carriage rates were similar at rural and urban trapping sites: 41 of the 118 small mammals (34.7%) caught in rural areas and 53 of the 150 animals (35.3%) caught in urban areas (p = 0.8). Regardless of the mammal species, infection rates were significantly higher in Moramanga (54%), Toliara (48%), and Mahajanga (47.4%) than in Antsiranana (8.5%) and Toamasina (14%) (p = 0.001). *Leptospira* carriage rates were significantly higher in *R. norvegicus* (48.9%), *S. murinus* (43.5%) and *R. rattus* (30.8%) than in *M. musculus* (9.1%) (p<0.001). There was also a trend towards higher infection rates in *R. norvegicus* than in *R. rattus* (p = 0.011).

**Table 2:** Prevalence of pathogenic *Leptospira* carriage in small mammals by site and species.

District	Diagnostic test		n positive/n analyzed (%)				
			*Rattus norvegicus*	*Rattus rattus*	*Mus musculus*	*Suncus murinus*	All
Moramanga	MAT		0/2	6/34	-	0/2	6/38 (15.8)
	PCR *hap1*	Kidney	3/3	19/34	1/10	2/3	25/50 (50)
		Urine	-	6/7	-	-	6/7 (85.7)
	Culture	Kidney	0/3	0/34	0/10	0/3	0/50 (0)
		Urine	-	0/7	-	-	0/7 (0)
	Any test		3/3 (100)	21/34 (61.8)	1/10 (10)	2/3 (66.7)	27/50 (54)
Antsiranana	MAT		0/5	0/29	0/2	-	0/36 (0)
	PCR *hap1*	Kidney	1/7	2/33	1/18	1/1	5/59 (8.5)
		Urine	0/2	0/3	0/4	0/1	0/10 (0)
	Culture	Kidney	0/7	0/33	0/18	0/1	0/59 (0)
		Urine	0/2	0/3	0/4	0/1	0/10 (0)
	Any test		1/7 (14.3)	2/33 (6.1)	1/18 (5.5)	1/1 (100)	5/59 (8.5)
Mahajanga	MAT		0/11	-	-	-	0/11 (0)
	PCR *hap1*	Kidney	15/42	-	0/4	7/13	22/59 (37.3)
		Urine	16/26	-	-	-	16/26 (61.5)
	Culture	Kidney	0/42	-	0/4	0/13	0/59
		Urine	0/26	-	-	-	0/26
	Any test		21/42 (50)	-	0/4	7/13 (53.8)	28/59 (47.4)
Toamasina	MAT		0/7	0/27	0/5	-	0/39 (0)
	PCR *hap1*	Kidney	1/9	6/27	0/8	0/6	7/50 (14)
		Urine	-	1/2	-	-	1/2 (50)
	Culture	Kidney	1/9	3/27	0/8	0/6	4/50 (8)
		Urine	-	1/2	-	-	1/2 (50)
	Any test		1/9 (11.1)	6/27 (22.2)	0/8 (0)	0/6 (0)	7/50 (14)
Toliaria	MAT		11/27	-	-	-	11/27 (40.7)
	PCR *hap1*	Kidney	19/35	-	3/15	-	22/50 (44)
		Urine	3/3	-	-	-	3/3 (100)
	Culture	Kidney	5/35	-	0/15	-	5/50 (10)
		Urine	0/3	-	-	-	0/3 (0)
	Any test		21/35 (60)	-	3/15(20)	-	24/50 (48)
All	Any test		47/96 (48.9)	29/94 (30.8)	5/55 (9.1)	10/23 (43.5)	91/268 (33.9)

DNA from pathogenic *Leptospira* strains was detected by *hap1* real-time PCR in 33.9% of the small mammals examined (Table 2). When available, urine samples gave a higher frequency of positive tests than did kidney samples (54.2% vs 30.2% p = 0.001) (Table 2). The control gene target was not amplified from three kidney samples that tested negative for *Leptospira* implying the presence of PCR inhibitors. The negative PCR results obtained for these samples were confirmed after 10-fold dilution of the extracted DNA samples as recommended by the manufacturer. The 16S rRNA gene (*rrs*) was amplified from 81 kidney or urine samples of the 107 testing positive by the *hap1* real-time PCR method. The amplification of 16S rRNA was mostly unsuccessful in *hap1*-positive samples demonstrating low levels of *Leptospira* DNA (Ct ranging from 37.9 to 40). We generated 333-nucleotide (nt) sequences from the 70 samples for which qualitatively satisfactory sequences were obtained; all were identical. *In silico* analysis identified this sequence as belonging to the species *L. interrogans*.

Positive MAT results were obtained for 11.3% (17/151) of the small mammals for which serum samples were available (Table 2). All 17 MAT-positive mammals were rats, but this finding might be biased because 94% of the serum samples were collected from rats, due to the ease of sampling from these species. All 17 MAT-positive mammals tested positive by *hap1*-based PCR and 15 also tested positive by *rrs*-based PCR. Seroprevalence was observed only in Moramanga and Toliara, in 15.8% and 40.7% of the animals, respectively (Table 2). All 17 positive sera showed reactivity against serogroup Canicola. Agglutination with serogroup Icterhaemorrhagiae occurred in 10 of these 17 sera (data not shown). In Moramanga, the reactivity of antibodies against the Icterohaemorrhagiae serogroup was stronger as evidenced by higher agglutination titers in the MAT than that of antibodies against the serogroup Canicola in four of the six samples and was similar in the other two (data not shown). Fresh isolates of the infecting serovar usually give higher titers when used as an antigen in MAT than the corresponding reference strain, which has undergone multiple passages *in vitro* [19], [20]. We subsequently included the local isolate TOA25R in the MAT analysis of sera from Toliara, to obtain clues as to the identity of the infecting leptospiral strain. TOA25R antigen was the most reactive antigen tested, giving the highest titer in 9 of the 11 (81.8%) positive samples, consistent with a major role of this isolate as an infectious agent. Seven samples tested positive for agglutinating antibodies against this local isolate only, indicating a higher overall seroprevalence than established with the reference panel

### Characterization of leptospiral isolates

**Figure 1:**
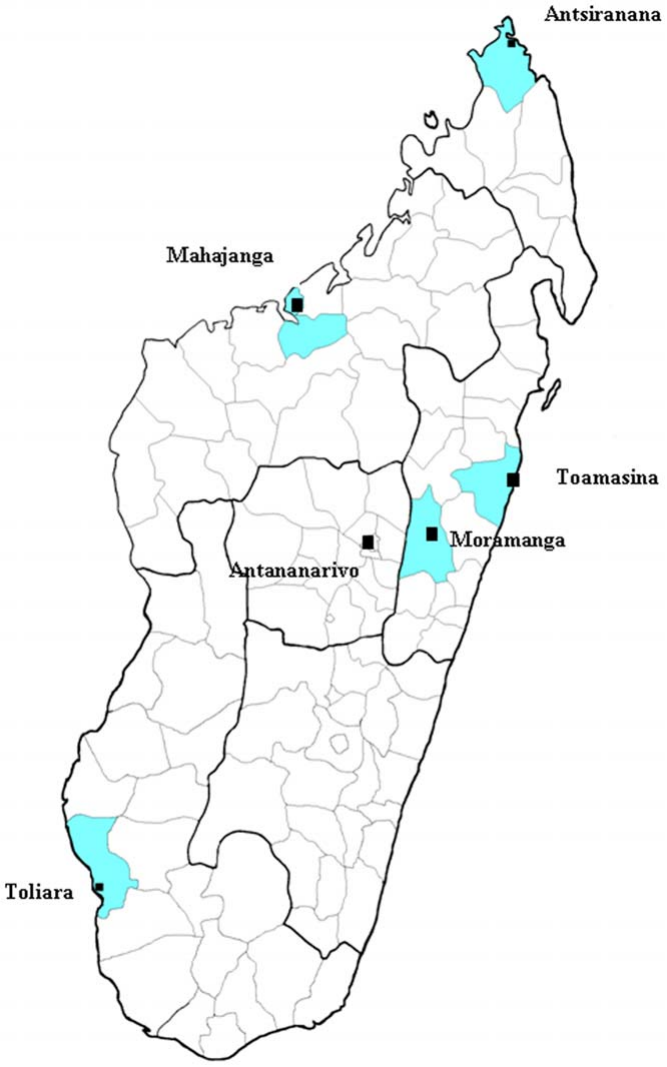
Map of Madagascar showing the five locations and districts (in blue) in which the field trapping survey was carried out. Square labels indicate cities.

Ten pathogenic *Leptospira* isolates were obtained from nine rats in the districts of Toamasina and Toliaria (Table 2). These contained isolates from kidney samples from three *R. rattus* captured within dwellings in the village of Fanandrana in the district of Toamasina (with confirmation by a positive urine culture in one case) and of one *R. norvegicus* captured in the harbor of Toamasina (Table 1 and Fig. 1). In the district of Toliara, cultures of samples from the kidneys of five *R. norvegicus* — three trapped in the urban area of Ankiembe and two in the harbor — yielded *Leptospira*. These isolates were named TOA13, TOA23, TOA25U, TOA25R, TOA44, TOL17, TOL24, TOL51, TOL54 and TOL55. TOA and TOL refer to the district — Toamasina and Toliara, respectively — the number is the mammal identification key and R or U indicates whether the isolate was obtained from the kidney or urine. All 10 culture-positive samples also tested positive by both *hap1* PCR (Ct of 23.9 to 34) and 16S rRNA PCR, suggesting that the *Leptospira* burden was high. The lengths of incubation required to obtain satisfactory growth ranged from two (TOA25R) to eight months (TOA 44 and TOL55) and did not correlate with the Ct values of the *hap1* PCR (data not shown).

**Figure 2:**
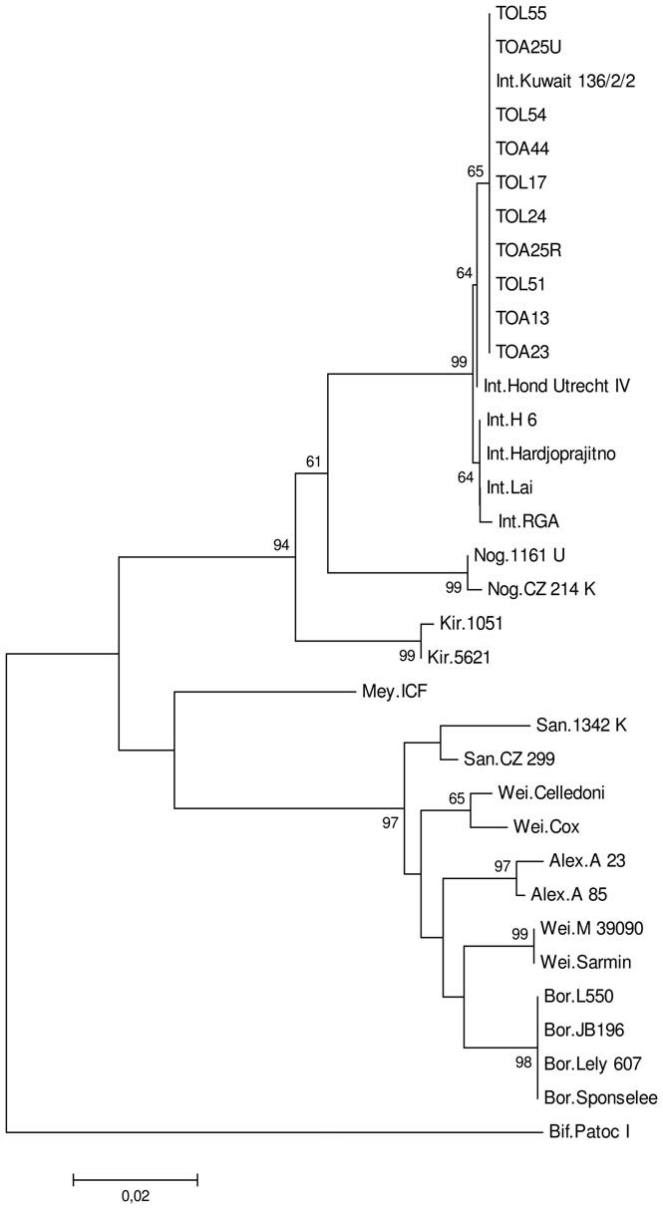
Phylogenetic tree based on Tamura-Nei distances and developed with the neighbor-joining method. Distances were calculated from G1–G2 restricted sequences and are based on 10 *Leptospira* strains isolated from rats in Madagascar (TOL55, TOL54, TOL51, TOL24, TOL17, TOA44, TOA25U, TOA25R, TOA23 and TOA13, GenBank accession numbers HM639728-HM639737). The remaining sequences are reference strains, including *Leptospira interrogans* (Int.) strains Lai, Hond Utrecht IV, Hardjoprajitno, Kuwait 136/2/2, H 6, RGA, *L.noguchii* (Nog.) strains CZ 214 K, 1161 U, *L. kirschneri* (Kir) 1051, 5621, *L.meyeri* (Mey.) strain ICF, *L.santarosai* (San.) strains 1342 K, CZ 299, *L.weilii* (Wei.) strains Celledoni, Cox, M 39090, Sarmin, *L. alexanderi* (Alex.) strains A 85, 23 A, *L. borgpetersenii* (Bor.) strains Sponselee, Lely 607, L550, JB196, and *L. biflexa*, as the outgroup (Bif.) strain Patoc I, (GenBank: EU358012.1, EU357961.1, EU357983.1, EU357970.1, EU357968.1, EU365950.1, EU365958.1, EU365957.1, EU357952.1, EU358015.1, EU365965.1, EU365956.1, EU358016.1, EU365960.1, EU358009.1, EU358065.1, EU365959.1, EU365963.1, EU365964.1, EU365954.1, EU365953.1, CP000348.1, CP000350.1 and EU365966.1 respectively). Numbers above branches represent the percentage of bootstrapping results (1000 replicates). Only bootstrap values above 50% are shown.

Serological typing with reference sera indicated that all isolates belonged to serogroup Canicola. Subsequent typing with a panel of Canicolagroup characteristic monoclonal antibodies (mAbs) revealed that all isolates corresponded to serovar Kuwait (Table 3). *secY* sequence-based phylogeny revealed that the sequences of all isolates were identical and, consistent with the serological typing, were identical to that of serovar Kuwait (Fig. 2).

**Table 3:** Identification of isolate TOA13 by comparison of its profile of MAT agglutination titers with a panel of mAbs characteristic for serovars in serogroup Canicola with those of reference serovars Kuwait, Canicola and Bafani.

	Isolate			
	Isolate TOA13	Kuwait	Canicola	Bafani
**mAb**				
F152C1	-	-	160	2560
F152C2	-	-	5120	5120
F152C5	-	-	5120	-
F152C7	-	80	5120	20480
F152C6	-	20	2560	320
F152C10	-	-	5120	2560
F152C11	2560	5120	10240	20480
F152C13	1280	1280	160	640
F152C14	-	20	1280	20480
F152C17	-	-	640	-
F152C18	-	-	1280	-

Numbers indicate reciprocal titers. TOA13 is shown as a typical example of all 10 isolates.

Two isolates, TOA13 and TOA25R, were selected at random for further characterization by PFGE and multi-locus VNTR analysis (MLVA). The PFGE fingerprints of these two isolates were identical, with one additional band compared to the pattern generated from the reference strain 136/2/2 of serovar Kuwait. The two MLVA patterns were also identical. VNTR-4, VNTR-7 and VNTR-10 amplifications each showed the same numbers of repeats, i.e. 0, 1 and 1, respectively. This corroborates with the specific pattern of strain 136/2/2 of serovar Kuwait [21]. Thus, both PFGE and MLVA results were consistent with the results of serological and phylogenetic analyses, confirming that the isolates are indeed *L. interrogans* serovar Kuwait.

These strains are stored in the collection of the WHO/FAO/OIE and National Collaborating Center for Reference and Research on Leptospirosis of the Royal Tropical Institute (KIT) (Amsterdam, The Netherlands) and in the collection of the National Reference Center for *Borrelia* (Institut Pasteur, Paris, France).

## Discussion

Our results provide the first direct evidence for the presence of *Leptospira* reservoirs in the rodent and shrew populations of Madagascar. Significant levels of *Leptospira* carriage in small mammals were demonstrated at five sites in this country, and the frequency of carriage was high at three of these sites (Moramanga, Mahajanga and Toliaria). Only one previous survey reported the possible existence of animal reservoirs and human infections [14]. However, no definitive proof was obtained by strain isolation and neither previous nor subsequent studies have confirmed these findings [15]–[17]. We have unambiguously demonstrated, by culture and PCR that kidney and urine infections occur in small animals on the island.

Our results, definitively demonstrating the presence of *Leptospira* sp. in Madagascar, contrast with previous studies, in which the presence of leptospirosis appeared doubtful. This may be explained by the design of our survey and the improved microbiological protocols we used. Firstly, we investigated a large sample of the small mammals regarded as the main reservoirs of this disease [1], [2]. Previous studies, if they included rodents at all, dealt with smaller samples analyzed with only one method (see Table S1 for a comparison of studies) [15], [16]. Secondly, our relatively broad distribution of trapping sites over the island provided better coverage of the heterogeneous distribution of the disease, which was found to have much higher prevalences in Moramanga, Toliara and Mahajanga than elsewhere. Toliara is the only site for which leptospirosis seroprevalence has previously been reported [14]. Thirdly, the timing of the five trapping sessions, over a two-year period, with at least three months between trapping campaigns, provided us with an opportunity to improve microbiological protocols. Positive culture results were obtained only during the second year of our study, through the use of better aseptic conditions during dissection, the crushing of kidney samples and sedimentation of the debris before mixing the supernatant with fresh medium. These measures reduced contamination and increased the likelihood of *Leptospira* being isolated from the medium. The other microbiological methods we used were also potentially more sensitive. Our results showed that the real-time PCR protocol was more sensitive than the conventional PCR method targeting the *rrs* gene, which was used in one of the previous studies [16]. Furthermore, the use of an internal control in our study revealed the presence of PCR inhibitors in part of the samples. This may account for the failure of PCR to identify leptospirosis in this previous study. The testing of urine samples is another probable advantage in our approach, because we confirmed the previously reported better performance of *hap*I amplification on urine samples than on kidneys in an animal model [18]. Finally, as discussed below, the use of more sensitive local isolates in the MAT serodiagnostic test appeared particularly informative. In addition to the impact of study methodology on the results, we cannot exclude the possibility of changes in the epidemiological profile of leptospirosis in Madagascar at this stage, although such changes are unlikely to have occurred in the short time period between this and the previous study [16].

The results of strain isolation in this study may have major implications for leptospirosis detection in Madagascar. The inclusion of a typical local isolate, TOA25R, in the MAT panel increased the rate of detection of specific antibodies. This finding is consistent with previous reports of an increase in MAT sensitivity associated with the use of local isolates [2], [19], [20]. The MAT is subject to a certain degree of serovar specificity and antigens vary considerably between regions [1], [2], [9]–[11], [20], [22], [23]. All 10 isolates were identified as *L. interrogans*, serogroup Canicola, serovar Kuwait. Higher positivity rates and the higher titers obtained by MAT including isolate TOA25R indicate that serovar Kuwait constitutes a major infectious agent within the small mammal populations investigated. Therefore a particular advantage from the isolates obtained in this study is that inclusion in diagnostic MAT panels imply a significant improvement of future leptospirosis detection in Madagascar.

Our results reveal considerable homogeneity among the 10 isolates in terms of both serological (serogroup Canicola mAbs) and molecular (*secY* phylogeny) characterization. Given the demonstrated discriminating power of *secY*-based phylogeny [24]–[26], [43], this uniformity is probably due to selection of the host species and geographic location. Indeed, *Leptospira* was isolated only from rat samples (*R. rattus* and *R. norvegicus*) collected in Toamasina and Toliara. The MAT results confirmed the low diversity of the serovars isolated from rats, with reactivity detected only against the serogroups Canicola and Icterhaemorrhagiae without excluding the possibility that agglutinations with serogroup Icterohaemorrhagiae are due to cross-reactions. There may be other serovars in other locations and the diversity of strains may be greater in other animal groups. Silverie *et al.* detected antibodies against other serogroups — Tarassovi and Bataviae — in cattle and swine [14] and a human infection on Mayotte (an island of the Comoros archipelago) due to a *L. kirschneri* serogroup Mini strain was recently suspected to have been acquired in Madagascar [27]. Further studies of more diverse reservoirs from different locations are required to complete this investigation of *Leptospira* epidemiology in Madagascar.

The diversity of leptospiral strains on the islands in close proximity to Madagascar has not been studied in detail, and only isolates from humans have been identified. On Mayotte, the epidemiological profile of leptospirosis is characterized by the scarcity of *L. interrogans* and the predominance of *L. borgpetersenii* and *L. kirschneri*, with Mini being the main serogroup [27]. On La Réunion, Icterohaemorrhagiae is the predominant serogroup, whereas, in the Seychelles, Icterohaemorrhagiae and Hurstbridge are the principal serogroups [9], [11], [28]. The situation in Madagascar reported here, with the predominance, if not exclusive presence, of *L. interrogans* serogroup Canicola serovar Kuwait, confirms the extensive differences between the *Leptospira* strains present on the various islands in this region of the Indian Ocean, contrasting with the apparently limited variation on each island.

This study did not include human infection, but the strains isolated in this study and the evidence obtained for the presence of significant small-mammal reservoirs may lead to public health programs for detecting human leptospirosis in Madagascar. We report, in at least three districts of Madagascar, a high prevalence (about 50%) of leptospiral carriage in small mammals. In other countries, rodent infection rates, determined on the basis of MAT, culture or conventional PCR protocols, have been reported to range from 10% in South Africa, 10% and 55% in Peru, 30% in Barbados and 46% in India to 80% in Brazil [20], [29], [30]–[34]. In these countries, the presence of major infection sources is associated with a high incidence of human leptospirosis. We hypothesize that this may also be the case for Madagascar. The small mammals of both rural and urban areas were highly infected, especially in the Moramanga, Mahajanga and Toliara districts. Thus, exposure to infection through field activities (rice farming), and through daily living activities in urban areas with poor living conditions, as observed in other developing countries, is likely [1], [2], [29], [30], [35]. Reports range from a virtual absence of leptospirosis [15], [16] to seropositivity rates of 51% in patients with clinically suspected leptospirosis. High rates of seropositivity suggest that leptospirosis transmission to humans occurs in Madagascar [14]. In addition to the composition of the MAT panel of serovars, low positivity rates may reflect environmental conditions affecting the survival of pathogenic *Leptospira* bacteria outside their host and, hence, the maintenance of infection reservoirs.

The prevalence of *Leptospira* carriage in small mammals was significantly higher in Moramanga, Mahajanga and Toliara, regardless of the species trapped, than in Antsiranana and Toamasina, which actually have higher annual rainfall levels. This situation appears paradoxical, as leptospirosis is usually considered to be favored by the tropical rainy climate, with outbreaks occurring during the rainy seasons or floods [1], [2]. In a previous study in Antananarivo, none of the 55 *Rattus rattus* or 50 *Pteropus rufus* analyzed was found to be carrying *Leptospira*. The authors hypothesized that this area, like others in Madagascar, may be less favorable for maintenance of the bacterium, because the environment is more acidic, with a low pH (<7.2) of freshwater bodies limiting leptospiral survival [1], [2], [15]. These authors suggested that other districts with higher water pH, such as the western coast of the island, should be explored. Consistent with this hypothesis, our results confirm the heterogeneity of leptospiral burden as a function of the district considered, with this burden possibly being higher on the west coast and in the highlands. However, it is not possible to assess the possible causal role of soil pH at this stage, because this factor was not investigated in this study.

The high level of leptospiral carriage observed in *R. norvegicus* (48.9%) may be associated with its aquatic habits. One of the common names of this species, the sewer rat, provides a clear indication of its favorite habitat. In Madagascar, despite being primarily an arboreal species secondarily adapted to houses, *R. rattus* is also encountered in rice fields. This behavior may account for the high prevalence of carriage in this species (61.8%) in the rural district of Moramanga. By contrast, domestic mice live mostly in dry parts of houses (rooms and food storage areas), resulting in lower levels of exposure to *Leptospira*. However, as we did not investigate the populations of mice living outdoors in the marshes, we have no conclusive evidence to suggest that the low level of leptospiral carriage found in this species is due to environmental factors [12]. Remarkably, *Rattus* sp. were found to be probable maintenance hosts for strains of serogroup Canicola, rather than those of serogroups Icterohaemorrhagiae and Ballum, which are more commonly associated with these reservoirs elsewhere [1], [2], [4], [23], [29]. This feature may be a key element specific to the epidemiology of leptospirosis in Madagascar. However, further studies including other mammal species are required to complete this analysis of leptospirosis reservoirs and diversity in Madagascar.

Serological assessment of the infection status of rodent populations has been shown to lead to underestimation. Natural hosts often have no antibodies against the commensal serovar because they are chronically infected and may become seronegative [32], [36], [37]. Consistently, negative MAT results were obtained for 72% of the rodents testing positive by PCR and 57.1% of those testing positive by culture (data not shown). The geographic restriction of seroprevalence to the Moramanga and Toliara districts remains unexplained.

Our results unambiguously demonstrate the presence of significant reservoirs of *Leptospira* in small mammals in Madagascar and highlight the urgent need for prospective clinical studies on leptospirosis in this country.

## Materials and Methods

### Small mammals: sites of capture, capture protocol and sample collection

Small mammal trapping campaigns were conducted in 2008/2009, in five districts of Madagascar described in Table 1 and Fig. 1. Moramanga, which is located at the edge of the central highlands, was investigated in April 2008, and the four coastal sites, Antsiranana, Mahajanga, Toamasina and Toliara, were surveyed in August 2008, November 2008, April 2009 and August 2009, respectively. These sites were selected on the basis of the natural history of the emergence or re-emergence of other infectious diseases, such as cholera and plague, in this country [38]–[40]. We hypothesized that leptospirosis might have been introduced via imported rodents carried by ships. At each site, small mammals were trapped in the city center and in neighboring rural areas. An additional location within the seaport facilities was also investigated in Antsiranana, Toamasina and Toliara (Table 1). The coordinates of the traps were determined with a global positioning system (GPS).

Small mammals were caught alive in wire-mesh traps (BTS, France) for rats, and aluminium Sherman traps for mice and shrews. These traps were left in place overnight on three consecutive nights. Within dwellings, both in the city center and in rural areas, we set up one BTS and one Sherman trap per house. The outdoor trapping scheme used in the seaports and in the rural areas consisted of a line of 20 traps (10 BTS and 10 Sherman traps in the seaport and 20 BTS at rural sites), spaced at 10 m intervals. In rural areas, these traps were placed along sisal hedges, which are used to pen in cattle or to protect gardens, and in rice fields. Captured animals were anesthetized with diethyl ether and blood was collected by cardiac puncture. The mammals were then killed and standard body measurements and weight were recorded. The animals were identified to species level. The kidneys were removed under aseptic conditions, and urine samples (when available) were collected by direct bladder puncture. All the protocols for animal trapping and use were in accordance with the guidelines of the Pasteur Institute of Antananarivo for animal handling and experiments. Here, these protocols did not require any approval by an ethics committee or wildlife administration service, as this study included only commensal, non endemic, rodents and shrews.

### Procedures

#### Isolation in culture and identification

One kidney from each mouse or shrew and half of a kidney from each rat was excised for culture. Inoculation in 9 ml of Ellinghaussen-McCullough medium as modified by Johnson & Harris (EMJH) (BioRad, Marnes la Coquette, France), supplemented with 5-fluorouracil (100 µg/ml) and rifampicin (10 µg/ml; Sigma-Aldrich, Lyons, France) was initially carried out without crushing the tissues. However, in the face of a high frequency of bacterial contamination and an absence of positive cultures, we adapted the protocol as follows, after the first year of the study. Kidney samples from small mammals captured in the Toamasina and Toliara districts were removed by an improved aseptic protocol in which the devices used were re-sterilized after cutting the skin. The kidney samples were then finely ground and used to inoculate 9 ml of EMJH medium. The homogenate was allowed to sediment for 10 minutes, and 1 ml of the debris-free supernatant was then mixed with 9 ml of EMJH fresh medium supplemented with antibiotics. When urine samples were available, we used four drops of urine to inoculate 9 ml of EMJH medium supplemented with antibiotics as indicated above. Cultures were incubated at 28°C and screened weekly by dark-field microscopy, for three months. During this three-month period of incubation, the medium was passed through 0.22 µm Millipore filter if contamination was suspected. If *Leptospira* growth was detected, 1 ml of the medium was mixed with 5 ml of fresh EMJH media without antibiotics for enrichment. The culture was subjected to the same procedure every month until a satisfactory density of bacteria was obtained. For some isolates, this process continued for eight months before satisfactory levels of growth were attained.

All the *Leptospira* isolates were sent to the WHO/FAO/OIE and National Leptospirosis Reference Centre, KIT Biomedical Research, Amsterdam, The Netherlands, for identification by serology and sequencing followed by phylogeny analysis.

For the identification of isolates to serogroup level, MAT was performed according to a standard procedure, with a panel of 43 rabbit anti-*Leptospira* antibodies [41]. Isolates were subsequently typed to serovar level by performing MAT with a panel of monoclonal antibodies (mAbs) characteristically agglutinating serovars from the serogroup Canicola (F152C1, F152C2, F152C5, F152C7, F152C8, F152C10, F152C11, F152C13, F152C14, F152C17 and F152C18), as previously described [42].

For molecular characterization *secY* sequence-based phylogeny analysis was carried out as previously described [25]. The phylogenetic power of *secY* has been convincingly demonstrated [24]–[26], [43]. Briefly, *Leptospira* was propagated at 30°C in EMJH liquid media without supplements. Genomic DNA was subsequently extracted with the QIAamp DNA extraction kit (Qiagen, GmbH, D-40724 Hilden, Germany), in accordance with the manufacturer's instructions. The DNA was then amplified and sequenced as described elsewhere [25]. DNA sequence alignments were generated with Vector NTI 10 software (Invitrogen). Phylogenetic analysis was conducted with MEGA 4.1 software [44]. One thousand bootstrap replications were used to assess the degree of confidence to be placed in the nodes. The tree was constructed by the Neighbor-Joining method in Jukes-Cantor mode [44].

Two isolates were randomly selected and blindly identified at the National Reference Center for Leptospirosis (Institut Pasteur, Paris, France), by agglutination with reference rabbit antisera, BLAST analysis of the 16S rRNA gene, pulsed-field gel electrophoresis (PFGE) after *Not*I restriction for serogroup, *Leptospira* species and strain determination, respectively, as previously described [45]. In addition, multi-locus VNTR analysis (MLVA) was used to confirm identification to the strain level, as previously described [21].

#### Molecular detection

The DNEasy Blood and Tissue Kit DNA (Qiagen, Courtaboeuf, France) was used to extract DNA from an amount of kidney material equivalent to that used for culture. Specimens were stored, in Eppendorf tubes containing 180 µl of ATL buffer at room temperature for a maximum of four days before extraction. We then added a further 180 µl of ATL and followed the manufacturer's instructions for DNA extraction. For DNA from urine, a few drops of urine were dispensed into an Eppendorf tube containing 200 µl of ATL buffer and the manufacturer's protocol was applied. DNA concentration and purity were assessed with a Nanodrop ND-1000 spectrophotometer (PEQLAB Biotechnologie GmbH). Samples were stored at +4°C until use or at −20°C for long-term storage.

For the detection of *Leptospira* DNA, we used a commercial real-time PCR kit (Adiavet® Lepto Realtime Kit; AES chemunex-Adiagène, Bruz, France). This kit specifically amplifies the hemolysis-associated protein 1 (*hap1/lipL32*) gene, which is present only in pathogenic *Leptospira* strains. An internal control DNA is included in each reaction, for the validation of negative results [18]. This test uses primers and TaqMan probes labeled with FAM for the *hap1* gene and VIC for the internal control. For each sample, 2 µl of DNA extract (from kidney or urine) was added to 23 µl of PCR mixture. The reaction mixtures were then incubated for 2 min at 50°C, 10 min at 95°C and subjected to 45 cycles of denaturation for 15 s at 95°C and annealing and elongation for 1 min at 60°C (StepOne, Applied Biosystems, Les Ullis, France). Samples presenting a typical amplification curve (a linear increase followed by a plateau) and a threshold cycle (Ct) value of less than 40 were considered positive for FAM or VIC. Positive and negative control reactions were included in each run, according to the manufacturer's instructions. A sample was considered negative if amplification was observed with VIC but not with FAM. A potential false-negative result due to the inhibition of PCR was recorded if no amplification was observed for either FAM or VIC. In such cases, the test was repeated with DNA diluted tenfold in sterile water, as recommended by the kit manufacturer. We then subjected samples testing positive for *hap1* by PCR to partial *rrs* amplification and sequencing, as previously described [45].

#### Serological analysis

Serum was isolated from whole-blood samples, dispensed into aliquots and stored at +4°C until processing. Serological testing was carried out in Frank Duncombe's laboratory (Caen, France), with the MAT reference method [46] and a panel of 12 live strains of *Leptospira* (serogroup, followed by serovar in brackets): Australis (Australis), Autumnalis (Autumnalis), Ballum (Castellonis), Bataviae (Bataviae), Canicola (Canicola), Grippotyphosa (Grippotyphosa), Icterohaemorrhagiae (Icterohaemorrhagiae and Copenhageni), Pomona (Pomona), Pyrogenes (Pyrogenes), Sejroe (Sejroe), Tarassovi (Tarassovi). A positive MAT result was defined as a titer ≥100 for at least one serovar of the panel. The serogroup giving the highest agglutination titer was considered to be the etiological agent. TOA25R, one of the first local strains obtained in this study, from Toamasina, was subsequently added to the panel for the testing of samples collected in Toliaria. The sera collected from the other four sites were entirely used up before the local strains were isolated and could not therefore be retested.

#### Statistical analysis

The study population was described separately for each district and each species. Qualitative variables are expressed as percentages. Groups were compared by χ^2^ tests or Fisher's exact test for categorical variables. A p value less than 0.05 was considered to denote statistical significance. If a significant difference between the four species was detected in these tests, pairwise comparisons were performed, with Bonferroni correction for multiple testing (a p value <0.008 (0.05/6) was consider to denote statistical significance). Data were analyzed with STATA software version 10.0 (Stata Corporation, College Station, Texas).

## Supporting Information

Table S1 Characteristics of studies conducted in Madagascar.
(0.04 MB DOC)
