# Supplementary material for: First Isolation and Direct Evidence for the Existence of Large Small-Mammal Reservoirs of Leptospira sp. in Madagascar
Source: PLoS One. 2010 Nov 24;5(11):e14111. doi: 10.1371/journal.pone.0014111 (PMC2991340; doi:10.1371/journal.pone.0014111)
Supplement: Table S1 — Characteristics of studies conducted in Madagascar. (0.04 MB DOC) [file pone.0014111.s001.doc]

Table S1: Characteristics of studies conducted in Madagascar

| **Study (Ref)** | **Population investigated (N)** | **Location** | **Study period** | **Methods** | | |
| --- | --- | --- | --- | --- | --- | --- |
|  |  |  |  | MAT | Culture | PCR / protocol |
| Brygoo *et al.*  (17) | Suspected humans (40)  Animals (168) ^a^ | Not specified | 1955 | Yes | No | No |
| Silverie *et al*. (14) | Suspected humans (65)  cattle (72), swine (25) | Toliara | 1968 | Yes | No | No |
| Lhuillier (15) | Non suspected humans (2646) | Antananarivo | 1978 | Yes | No | No |
|  | *R. rattus* (55), *Pteropus rufus* (50) | Marovitsika-Anjiro |  | No | Yes  (1 month of incubation) | No |
| Ralaiarijaona *et al.* (16) | Non suspected humans ^b^ (105) | Antananarivo | 2000 | Yes | No | No |
|  | *R. rattus* (49) , *R. norvegicus* (66), cattle ( 50), swine (13) | Antananarivo, Ambositra, |  | No | No | Yes / Conventional without internal control |
| Rahelinirina *et al*. (this study) | *R. rattus* (94), *R. norvegicus* (96), *Mus musculus* (55), *Suncus murinus* (23) | Moramanga, Antsiranana, Mahajanga, Toamasina, Toliara | 2008-2009 | Yes (using local isolates once available) | Yes  (3 months of incubation | Yes / real-time with internal control |

^a^ 24 dogs, 10 donkeys, 20 horses, 49 swine, 65 cattle

^b^ Occupationally exposed
